# A double whammy: The association between comorbidities and severe dengue among adult patients—A matched case-control study

**DOI:** 10.1371/journal.pone.0273071

**Published:** 2022-09-20

**Authors:** Wei Yao Ng, Rafidah Atan, Nor’azim Mohd Yunos, Adam Harrish bin Md Kamal, Mohd Hariz Roslan, Kai Yuan Quah, Kai Xuan Teh, Masliza Zaid, Mahazir Kassim, Jeevitha Mariapun, Chin Fang Ngim, Amreeta Dhanoa, Tsin Wen Yeo

**Affiliations:** 1 Jeffrey Cheah School of Medicine and Health Sciences, Monash University Malaysia, Selangor, Malaysia; 2 Mackay Base Hospital, Mackay Hospital and Health Services, West Mackay, Queensland, Australia; 3 Department of Anesthesiology, Faculty of Medicine, Universiti Malaya, Kuala Lumpur, Malaysia; 4 Department of Medicine, Faculty of Medicine, Universiti Malaya, Kuala Lumpur, Malaysia; 5 Department of Medicine, Hospital Sultanah Aminah, Johor Bahru, Johor, Malaysia; 6 Department of Anesthesiology and Intensive Care, Hospital Sultanah Aminah, Johor Bahru, Johor, Malaysia; 7 Lee Kong Chian School of Medicine, Nanyang Technological University and Imperial College, Singapore, Singapore; James Cook University, AUSTRALIA

## Abstract

**Background:**

Dengue infection is the most prevalent mosquito-borne viral infection globally. Concurrently, there has also been an upsurge of non-communicable comorbidities. We aimed to investigate the association between these comorbidities and the development of severe dengue.

**Methods:**

We performed a retrospective, case-control study involving 117 cases with severe dengue and 351 controls with non-severe dengue; matched according to gender, age (+/- 5 years old), and admission date (+/- 2 weeks). We analyzed the data using conditional odds ratio (cOR) and adjusted conditional odds ratio (AcOR) using univariate and multivariable conditional logistic regression respectively.

**Results:**

Six main comorbidities namely obesity, diabetes mellitus, hypertension, hyperlipidemia, chronic pulmonary disease, and ischemic heart disease were observed among cases and controls. Multivariable conditional logistic regression model found only hypertension to be independently associated with the development of severe dengue (ACOR 2.46; 95% CI:1.09–5.53). Among symptoms at presentation, lethargy, vomiting, bleeding manifestations, and abdominal pain were associated with increased odds of severe dengue, although the associations were not statistically significant. Headache (ACOR: 0:32; 95% CI: 0.21–0.51) and skin rash (ACOR: 0.42; 95% CI: 0.22–0.81) were associated with significantly lower odds of severe dengue. Severe dengue patients were also found to have significantly higher white cell count, urea, creatinine, alanine aminotransferase, aspartate aminotransferase, creatine kinase, and lactate dehydrogenase on admission, while platelet and albumin were significantly lower compared to non-severe dengue patients.

**Conclusions:**

Our study found a significant association between hypertension and the development of severe dengue in adult patients. For clinical practice, this finding suggests that dengue patients with underlying hypertension warrant closer clinical monitoring for deterioration. The association between significant derangement in various laboratory parameters and severe dengue as shown in this study is in keeping with previous reports. While further substantiation by larger prospective studies will be desirable, this association may serve to inform the dengue triaging process.

## Introduction

Dengue is an acute viral illness caused by the Dengue virus (DENV), a single-stranded RNA virus belonging to the family *Flaviviridae*, with four different serotypes (DENV-1, DENV-2, DENV-3, and DENV-4) [1]. It is transmitted by the *Aedes* mosquitoes and it is endemic in more than 100 countries across Southeast Asia, Western Pacific, and the Americas. Globally, the number of dengue cases has quadrupled in three decades since 1990. In a systematic analysis by Zhilin Zeng et al, the authors highlighted that dengue cases rose from 23 283 274 (around 23 million) in 1990 to 104 771 911 (around 104 million) in 2017; with an in a tandem increase in the incidence of deaths from around 16 957 cases in 1970 to 40 467 cases in 2017 [2]. In terms of the financial burden of dengue, the total annual global cost of managing dengue illness was estimated at USD 8.9 billion [3].

Clinically, dengue has a wide spectrum of presentations ranging from an asymptomatic self-limiting course to life-threatening complications such as shock, hemorrhagic manifestations, and organ failure. To classify the severity of dengue cases, the 2009 WHO classification divides patients into dengue without warning signs, dengue with warning signs, and severe dengue. A patient infected with dengue can be diagnosed with severe dengue if the patient fulfills one of these three criteria: severe plasma leakage, severe bleeding, or severe organ involvement [4]. The 2009 revision is an improvement over the 1997 WHO classification [5] which failed to capture organ failure [6] and was impractical, as revealed by the landmark DENCO trial where 22% of dengue patients complicated with shock were unable to fulfill all four criteria to be diagnosed as dengue hemorrhagic fever (DHF) [7].

Dengue is believed to be a complex process involving the interaction between the immune system, host factors, and the DENV [8]. This is corroborated by early epidemiological studies that pointed out that the tendency to develop DHF or Dengue Shock Syndrome (DSS) was higher amongst patients with secondary dengue infection of a different serotype [9, 10]. Halstead [9, 10] hypothesized that when a previously infected individual is subsequently infected by either of the other three DENV serotypes, pre-existing antibodies can bind to the “foreign DENV serotype” to form antibody-virus complexes causing a more severe course of the disease [11]. More recently, non-communicable comorbidities have also been implicated in the development of DHF, DSS, or severe dengue. A matched case-control study conducted in Brazil found that the odds of progressing into DHF were 2.75 times higher among dengue patients with diabetes mellitus [12]. Another matched case-control study, also from Brazil, reported that the odds of developing DHF were 1.60 times higher among dengue patients with underlying hypertension [13]. These findings are corroborated by a retrospective case-control study performed in Singapore which reported that the odds of developing DHF were significantly higher amongst dengue patients with underlying diabetes mellitus as well as dengue patients with diabetes mellitus and hypertension [14]. A matched-case-control in Pakistan showed that the odds of developing DHF were higher amongst those with diabetes mellitus or hypertension, though the findings failed to attain statistical significance [15]. These associations seem plausible as patients with these comorbidities have been linked with a host of dysregulated immune responses, metabolic derangement, and perturbations at the endothelial layer [16] which could play a role in aggravating the heightened immune response and intractable endothelial hyperpermeability seen in severe dengue. It is reported that 63% of adults in Malaysia aged 18 years and above have at least one of the four non-communicable comorbidities: obesity, hypertension, diabetes mellitus, or hyperlipidemia [17]. Dengue is also hyperendemic in Malaysia. In 2017 alone, there were 83,849 cases of dengue reported nationwide with 177 cases which resulted in mortality [18]. A meta-analysis on the pediatric population found obesity was significantly associated with the development of DHF and DSS [19]. However, another retrospective cohort study in Malaysia failed to demonstrate obesity as a significant factor associated with the development of severe dengue [20]. Zulkipli et al in their meta-analysis recommended that more studies should be conducted to evaluate the association between obesity and dengue severity in the adult population before any conclusions can be drawn [19].

Considering these conflicting reports, we conducted a case-control study in which the primary objective is to investigate the association between underlying medical comorbidities with severe dengue. The secondary objective is to further elucidate the association between clinical presentation and admission laboratory parameters with severe dengue.

## Methods

A retrospective, matched case-control study was conducted to compare cases of severe dengue to controls admitted to Hospital Sultanah Aminah Johor Bahru (HSAJB) from January 2017 to April 2019. HSAJB is the main tertiary 1000-bedded public hospital in Southern Malaysia with well-equipped facilities. All patients admitted with dengue are managed using a dengue care pathway stipulated by our national clinical practice guidelines.

### Inclusion criteria

(a) Cases: adult patients (aged 18 years and above) who fulfilled the diagnosis of severe dengue, defined by the WHO 2009 Dengue Classification. For further clarification, all severe dengue patients were managed in the intensive care unit at our study site, HSAJB as per the local hospital’s protocol. (b) Controls: adult patients (aged 18 years old and above) with a diagnosis of non-severe dengue, who did not progress to severe dengue throughout the hospitalization period. At our study site, such non-severe dengue patients were managed in a dedicated general medical ward known as the” Hibiscus Ward”

### Exclusion criteria

We excluded dengue patients who were pregnant.

The diagnosis of dengue was made based on either laboratory-confirmed dengue or probable dengue under the 2009 WHO Dengue case classification. Laboratory-confirmed dengue were those with positive NS1 results obtained via rapid dengue diagnostic kit commercially known as the SD BIOLINE Dengue Duo Kit (Standard Diagnostic Inc., Korea). Probable dengue patients had a clinical presentation fulfilling the WHO 2009 criteria for probable dengue, namely fever plus at least two of the following: Nausea or vomiting, rash, aches and pains, positive tourniquet test, leukopenia, and any warning signs.

In addition to clinical criteria, probable dengue patients must have either a supportive serology result (IgM or IgG) or a positive Dengue IgM antibody on a late-acute or a convalescent phase serum specimen. Dengue serology results (IgM or IgG) were obtained either from the SD Bioline Dengue Duo Kit or the PanBio Dengue IgM or IgG Capture ELISA (Standard Diagnostic Inc., Korea).

Severe dengue was defined under the WHO 2009 guidelines [7] with some slight modification [6, 19, 20]. A dengue patient is diagnosed with severe dengue if he/she develops at least one of three criteria:

1. Severe plasma leakage leading to shock or fluid accumulation with respiratory distress.
2. Severe bleeding is defined by either a clinician’s judgment of severe bleeding, life-threatening bleed such as hematemesis, melaena, or intracranial bleed, or bleeding that gives rise to hemodynamic instability necessitating fluid replacement for shock and/or whole blood or packed cell transfusion.
3. Severe organ involvement which includes either:
  (a) Severe liver impairment (AST or ALT ≥ 1000 IU/L)
  (b) Central nervous system impairment (encephalopathy)
  (c) Cardiac impairment (myocarditis)

and/or

(d) Acute kidney injury (AKI): defined according to the Kidney Disease: Improving Global Outcomes (KDIGO) guidelines as any of the following: rise in serum creatinine by ≥26.5μmol/l or more within 48 hours; or rise in serum creatinine to ≥1.5 times baseline (known or presumed to have occurred within the last 7 days); or urine output of <0.5ml/kg/h for 6 hours

The Medical Research & Ethics Committee of the Ministry of Health Malaysia provided approval for this study with approval number of NMRR-19-17-45660. As this study was retrospective in nature and patients’ information was analyzed anonymously, informed consent from patients was waived.

### Selection and matching of controls

A case-control ratio of 1:3 was decided to increase precision while considering the element of feasibility. Controls were patients admitted to the general medical ward who matched the case in terms of gender, age group (matched within 5 years of the case’s age), and hospital admission date (matched within 2 weeks of the patient’s date of admission). When more than three potential controls were available for a particular case, up to ten suitable controls were selected and randomized using the randomization tool in Microsoft Excel 2016 to select the top three controls to minimize selection bias. The limit to 10 potential controls was due to feasibility issues. The compilation of the list was done manually and involved tracing of patients using census records. For a minority of cases (less than 5 percent), for which there was a lack of at least 3 suitable controls that met the criteria for matching, the age group was expanded to within 10 years of the index case’s age.

Matching for age and sex was conducted to account for possible differences in outcome between different age groups and sex. Matching according to hospital admission dates was also deemed necessary to avoid confounding in terms of variation in circulating dengue serotypes. The list of these cases and controls were subsequently compiled and sent to the hospital’s record office to retrieve the patient’s original inpatient case notes.

### Data collection

All clinical data were collected retrospectively from the patient’s original medical case notes and hematology and biochemical laboratory charts. Information extracted included demographic data (age, gender, ethnicity, nationality, height, weight), comorbidities, presenting signs and symptoms, and hematological and biochemical laboratory parameters on admission. Comorbidities traced from records included obesity, diabetes mellitus, hypertension, hyperlipidemia, chronic kidney disease, chronic pulmonary disease, and stroke. Obesity was defined as a body mass index (BMI) of ≥ 27.5 kg/ m^2^, calculated based on admission data. This complies with WHO’s recommended classification for adults in Asian populations and also adopted by the Malaysian’s Clinical Practice Guideline for obesity [21]. Chronic pulmonary disease included those with asthma or chronic obstructive pulmonary disease. Other comorbidities were recorded according to formal diagnoses ascribed to respective patients in the notes.

Presenting signs and symptoms included duration of fever, abdominal pain, diarrhea, vomiting, lethargy, musculoskeletal (MSK) symptoms (myalgia, arthralgia or bone pain), chills or rigors, upper respiratory tract infection (URTI) symptoms (runny nose, sore throat or cough), bleeding manifestations (gum bleed, epistaxis, hemoptysis, hematemesis, melaena or vaginal bleed), headache and skin rash. Hematological laboratory results on admission included hemoglobin (Hb), hematocrit (Hct), white cell count (WCC), and platelet count while biochemical laboratory results comprised of urea, creatinine, alanine aminotransferase (ALT), aspartate aminotransferase (AST), albumin, creatine kinase (CK), lactate dehydrogenase (LDH). The data were recorded in a case report form (CRF) to facilitate uniform data recording.

### Sample size

The required sample size was calculated using Sampsize, an online sample size calculator for case-control studies [22]. Based on a power of 80%, 95% confidence interval, an expected minimum odds ratio (OR) of 2.0, a ratio of 3 controls per case, and percentage of exposed controls set at 18%, we determined that we would require 472 patients consisting of 118 cases and 354 controls. The percentage of exposed controls was set at 18% as this is the prevalence of diabetes mellitus among Malaysia’s adult population, which is also the comorbidity with the smallest prevalence among the comorbidities being studied) [17]. The odds ratio of 2.0 was decided based on a previous study by Badawi et al who reported the odds ratio of severe dengue was around 2 to 4 for patients with comorbidities such as diabetes, hypertension, and heart disease.

### Statistical analysis

Statistical analyses were performed via Stata IC 15.0 (StataCorp LLC ®) [23]. Differences in demographics, comorbidities, presenting signs and symptoms, and admission laboratory parameters were compared between cases and controls. Categorical variables were expressed in numbers and percentages while continuous variables were expressed in median and interquartile range(IQR) as data were not normally distributed. The normality of continuous data was evaluated using the Kolmogorov-Smirnov test. For descriptive analyses, categorical variables were compared using either the Pearson’s chi-square (χ2) or Fisher’s exact test. Most continuous variables collected were non-normal in distribution and were analyzed using the Wilcoxon rank-sum test (Mann-Whitney U test). Being a matched case-control study, conditional logistic regression was utilized to factor in the matching criteria used in our study (age, gender, admission date). Conditional odds ratio (cOR) and adjusted conditional odds ratio (AcOR) were analyzed using univariate and multivariable conditional logistic regression respectively. The confounding effect was further minimized using multivariable conditional logistic regression adjusting for potential confounders identified in the univariate analysis. Potential confounders were variables on descriptive analysis (Table 1) with statistically significant differences (p<0.05) between the cases and controls. All tests were performed at the 5% level of significance with a conditional odds ratio(cOR) or adjusted cOR(AcOR), p-value, and 95% confidence interval(CI) shown in tables, respectively. Model fitness of the multivariable conditional logistic regression model was determined with the Hosmer-Lemeshow test.

**Table 1:** Baseline Characteristics of cases (severe dengue) and controls (non-severe dengue).

Baseline Characteristics	Cases (Severe dengue) (*n* = 117)		Controls (Non-severe dengue) (*n* = 351)		p-value^c^
	N	(%)	N	(%)	
**Severe dengue manifestations** ^a^					
**Fluid accumulation leading to shock or respiratory distress**	**60**	**68.4**			
**Severe bleeding**	**33**	**28.2**			
**Severe organ impairment**	**71**	**60.7**			
**Age(years)** ^b^					
**median (Interquartile range)**	40	(29–49)	38	(28–48)	0.531^d^
**Gender** ^a^					
**Male**	57	48.7	171	48.7	1.000
**Female**	60	51.3	180	51.3	
**Ethnicity** ^a^					
**Malay**	50	42.8	159	45.3	0.961
**Chinese**	17	23.9	82	23.4	
**Indian**	28	14.5	50	14.2	
**Others**	22	18.8	60	17.1	
**Citizenship** ^a^					
**Resident**	96	82.1	285	81.2	0.837
**Foreigner**	21	21.9	66	18.8	
**Laboratory-confirmed(L) or Probable(P) Dengue** ^a^					
**L**	94	80.3	225	78.3	0.647
**P**	23	19.7	76	21.7	
**Dengue IgG Seropositivity** ^a^ *					
**Positive**	40	34.2	139	39.6	0.297
**Negative**	77	65.8	212	60.4	
**Obesity** ^a^	45	38.5	100	28.5	0.043
**Diabetes Mellitus** ^a^	23	20.2	35	10.0	0.006
**Hypertension** ^a^	31	26.5	42	12.0	<0.0001
**Hyperlipidemia** ^a^	12	10.3	15	4.3	0.016
**Chronic Pulmonary Disease** ^a^	6	5.1	12	3.4	0.410^e^
**Ischemic Heart Disease** ^a^	4	3.4	7	2.0	0.479^e^

^a^ Categorical variable; expressed as number (percentage)

^b^ Continuous variable; expressed as median (interquartile range)

^c^ Pearson chi-square, unless specified otherwise

^d^ Mann-Whitney U test

^e^ Fisher’s exact test

* Dengue IgG seropositivity is taken as a surrogate marker for secondary dengue infection

## Results

A total of 117 cases and 351 controls admitted to our study site (HSAJB) from January 2017 to April 2019 were included in this study. Being a case-control study matched by gender and age with a case-to-control ratio of 1:3, there were 57 males and 60 females among the cases with the corresponding 171 males and 180 females in the control group. The median age of the cases was 40 years old while the median age of the controls was 38 years old. In terms of ethnicity of the cases, the majority were Malays (43%), followed by Chinese (24%) and Indians (15%). A similar trend was also noted among the control group in which 45% of them were Malays with Chinese and Indians making up 23% and 14% respectively. No statistically significant differences were observed between the cases and controls in terms of gender, age, and ethnicity (p>0.05). Analysis of Dengue IgG seropositivity also found no significant difference between the cases and controls (p = 0.297), implying no significant difference in secondary dengue infection between the cases and controls. A total of six different comorbidities were compared, namely obesity, diabetes mellitus, hypertension, hyperlipidemia, chronic pulmonary diseases, and ischemic heart disease (Table 1). Out of these comorbidities, the proportion of obesity, diabetes mellitus, hypertension, and hyperlipidemia was significantly higher among the cases compared to the controls (p<0.05).

The univariate conditional logistic regression analysis identified, obesity (cOR: 1.57; 95% CI: 1.01–2.43), diabetes mellitus (cOR: 2.21; 95% CI:1.24–3.92), hypertension (cOR:2.65; 95% CI:1.57–4.47) and hyperlipidemia (cOR:2.56; 95% CI:1.16–5.64) to have statistically significant association with severe dengue (Table 2). These four comorbidities were then adjusted for in the conditional logistic regression model. The adjusted model found that those with hypertension had a statistically significant increased odds of severe dengue (AcOR:2.46; 95% CI:1.09–5.53).

**Table 2:** Conditional odds ratio (cOR) and adjusted conditional odds ratio (AcOR) for the association between comorbidities and severe dengue.

	cOR	95% CI	p value (cOR)	AcOR*	95% CI	p value (AcOR)
**Obesity**	1.57	1.01–2.43	0.043	1.45	0.89–2.34	0.132
**Diabetes Mellitus**	2.21	1.24–3.92	0.006	1.68	0.74–3.78	0.212
**Hypertension**	2.65	1.57–4.47	<0.0001	2.46	1.09–5.53	0.029
**Hyperlipidemia**	2.56	1.16–5.64	0.020	1.38	0.49–3.88	0.538

* AcOR was obtained from a multivariable conditional logistic regression model being adjusted by obesity, diabetes mellitus, hypertension, and hyperlipidemia. Model fitness was tested with the Hosmer-Lemeshow test (p = 0.820). The final model has an overall correct classification of 75.0% AcOR, adjusted conditional odds ratio; CI, confidence interval; cOR conditional odds ratio.

Results comparing various clinical presentations on admission between the cases and controls are shown in Table 3. Using univariate analysis, the odds of developing severe dengue were significantly higher in dengue patients who presented with lethargy (COR: 1.89; 95% CI: 1.08–3.30); and significantly lower in dengue patients who presented with headache (COR: 0.34; 95% CI: 0.22–0.53) and skin rash (COR: 0.45; 95% CI: 0.24–0.85). Following adjustment in the multivariable conditional logistic regression model, only headache and skin rash remained to be statistically significant. The odds of developing severe dengue were significantly lower among those who presented with headache (AcOR:0.32; 95% CI: 0.21–0.51) and skin rash (AcOR: 0.42; 95% CI: 0.22–0.81) respectively. There was no difference between patients with severe dengue and non-severe dengue in terms of presentations such as abdominal pain, diarrhea, vomiting, lethargy, chills and rigors, URTI symptoms, bleeding manifestations, and musculoskeletal symptoms (Table 3).

**Table 3:** Clinical presentation on admission of cases (severe dengue) compared to controls (non-severe dengue).

Clinical presentation	Cases (n = 117)	Controls (n = 351)	cOR	95% CI	p value	AcOR*	95% CI	p value
**Signs and Symptoms**								
**Abdominal Pain**	49	134	1.18	0.76–1.83	0.455	1.18	0.75–1.85	0.483
**Diarrhea**	63	193	0.95	0.63–1.45	0.819	0.97	0.63–1.50	0.892
**Vomiting**	83	214	1.57	0.99–2.49	0.055	1.57	0.97–2.56	0.068
**Lethargy**	99	262	1.89	1.08–3.30	0.027	1.78	1.00–3.18	0.051
**Headache**	49	238	0.34	0.22–0.53	<0.0001	0.32	0.21–0.51	<0.0001
**Rash**	13	76	0.45	0.24–0.85	0.013	0.42	0.22–0.81	0.010
**Chills and Rigors**	21	59	1.09	0.62–1.93	0.765	1.04	0.57–1.92	0.889
**URTI Symptoms**	21	46	1.44	0.82–2.54	0.537	1.46	0.82–2.63	0.202
**Hemorrhagic Symptoms**	29	82	1.09	0.67–1.80	0.730	1.20	0.72–2.01	0.484
**MSK Symptoms**	86	268	0.85	0.52–1.39	0.515	0.83	0.49–1.39	0.472

%, percentage; AcOR, adjusted conditional odds ratio; CI, confidence interval; cOR, conditional odds ratio; IQR, interquartile range; MSK, musculoskeletal; URTI, upper respiratory tract infections

* AcOR was obtained from a multivariable conditional logistic regression being adjusted by obesity, diabetes mellitus, hypertension, and hyperlipidemia.

Results comparing laboratory parameters between the cases and controls are displayed in Table 4. On admission, patients with severe dengue have significant derangements in various hematological and biochemical laboratory parameters. Hematological investigations showed that patients with severe dengue have significantly higher white cell counts (median 5.00; IQR: 3.20–7.60) but significantly lower platelet counts (median 44; IQR 22–104) compared to non-severe dengue patients. No statistically significant differences were observed between the two groups in terms of their admission hemoglobin levels. As for biochemical investigations on admission, severe dengue patients were shown to have significantly higher levels of urea (median 5.50; IQR: 3.30–9.30), creatinine (median 87; IQR: 63–151), ALT (median 144; IQR: 46–527), AST (median 300; IQR: 86–1159), CK (median 216; IQR: 98–620) and LDH (median 659; IQR: 380–1433) and significantly lower serum albumin levels (median 34; IQR: 30–38; p<0.0001) compared to non-severe dengue patients.

**Table 4:** Admission laboratory parameters of cases (severe dengue) compared to controls (non-severe dengue).

Admission Lab Parameters	Cases (n = 117)	Controls (n = 351)	cOR	95% CI	p value	AcOR*	95% CI	p value
**Median White Cell Count (10** ^**9**^ **/L)**	5.0	3.50	1.31	1.19–1.44	<0.0001	1.30	1.18–1.43	<0.001
**Median Platelet (10** ^**9**^ **/L)**	44	73	0.99	0.99–1.0	0.003	0.99	0.99–1.0	0.007
**Median Hemoglobin (g/dL)**	14.4	14.3	1.04	0.93–1.16	0.538	0.99	0.88–1.11	0.868
**Median ALT (U/L)**	144	51	1.007	1.005–1.009	<0.0001	1.006	1.004–1.009	<0.0001
**Median AST (U/L)**	300	78	1.004	1.003–1.006	<0.0001	1.002	1.003–1.006	<0.0001
**Median Urea (mmol/L)**	5.5	3.3	1.43	1.28–1.59	<0.0001	1.41	1.27–1.58	<0.0001
**Median Creatinine (μmol/L)**	87	69	1.03	1.02–1.04	<0.0001	1.03	1.02–1.04	<0.0001
**Median Albumin (g/L)**	34	38	0.82	0.77–0.87	<0.0001	0.82	0.77–0.87	<0.0001
**Median LDH (U/L)**	659	348	1.004	1.002–1.005	<0.0001	1.004	1.002–1.005	<0.0001
**Median CK (U/L)**	216	165	1.001	1.0003–1.001	0.001	1.008	1.003–1.001	0.002

AcOR, adjusted conditional odds ratio; ALT, alanine aminotransferase; AST, aspartate aminotransferase; CI, confidence interval; CK, creatinine kinase; cOR, conditional odds ratio; IQR, interquartile range; LDH, lactate dehydrogenase.

* AcOR was obtained from a multivariable conditional logistic regression adjusted for obesity, diabetes mellitus, hypertension, and hyperlipidemia.

## Discussion

Our case-control study found an association between hypertension and severe dengue. Although the exact mechanism that links hypertension to a higher risk of severe dengue is not well understood, there are a few plausible explanations. Both hypertension and severe dengue involve overactivation of the host immune system [24, 25]. Hypertension is also linked to a pro-inflammatory state [24, 26] with studies demonstrating significant elevation of inflammatory markers such as interleukin-6 (IL-6) and c-reactive protein (CRP) in hypertensive subjects [26, 27]. This pro-inflammatory state in hypertensive patients has been linked to vascular endothelium dysfunction [28] which may, in turn, lead to manifestations of severe dengue.

Another possible link between severe dengue and hypertension is through their effects on the endothelial glycocalyx (EG) layer [29]. Various animal and human studies have observed EG layer disruption in comorbidities such as diabetes mellitus and hypertension. EG is a semi-permeable membrane lining the luminal side of the vascular endothelium and is crucial in the hemostatic control of fluid exchange between intravascular and extracellular spaces [30]. We found no human studies on the association between hypertension and EG disruption, however, an animal experiment showed that mean EG thickness of both retinal and choroidal capillaries were significantly reduced amongst hypertensive mice as compared to mice without hypertension [31]. Therefore, disruption of EG with resulting disruption to fluid homeostasis may be a contributing factor in the development of severe dengue in patients with diabetes mellitus and hypertension.

Our study demonstrated that the odds of severe dengue were significantly lower in those presenting with headache or skin rash. No significant differences were noted for abdominal pain, diarrhea, vomiting, lethargy, chills and rigors, URTI symptoms, hemorrhagic manifestations, and musculoskeletal symptoms. A recent meta-analysis showed that there were four symptoms significantly associated with elevated risks of severe dengue namely nausea, vomiting, bleeding (gum bleeding, epistaxis, hematemesis, melaena), abdominal pain, and skin rashes [32]. A retrospective cohort study in Singapore involving 82 DHF patients and 1855 DF patients also reported that headache on presentation was linked to lower odds of development of DHF [33]. Nevertheless, some caution should be exercised when interpreting these results as the mechanisms remain unclear.

Patients with severe dengue were noted to have significant derangements in various laboratory parameters. Thrombocytopenia is a common feature seen in acute dengue infection, believed to be caused by various closely linked mechanisms which include bone marrow suppression by DENV and pro-inflammatory cytokines generated during dengue infection [34] as well as immune mechanisms in which cross-reactive dengue antibodies interact with DENV antigens on the platelets, leading to lysis of platelets in the peripheral circulation through complement activation [35, 36]. In our study, severe dengue patients were found to have significantly lower platelet counts on admission.

In addition to thrombocytopenia, leukopenia (white cell count less than 4.00X10^9^/L) [37] is another typical laboratory feature seen in patients during the febrile phase secondary to DENV-induced bone marrow suppression. However, our study demonstrated that patients with severe dengue had significantly higher white cell counts (WCC) as compared to non-severe dengue patients on admission. The median WCC on admission for the non-severe dengue group indicated leukopenia (median 3.50X10^9^/L) while the median WCC on admission for the severe dengue group was above the leukopenia range (median 5.00X10^9^/L). Leukocytosis has been associated with severe dengue [38] and in fact, a WBC >5.00×10^9^/L has been shown to be a prognostic factor for dengue severity [39]. It has been postulated that a higher white cell count in more severe dengue cases may indicate bacterial co-infection or gastrointestinal bleeding [38, 40] and should caution clinicians about a potential deterioration in dengue patients.

Severe dengue is also known for causing severe impairment in various organs, most commonly the liver, kidney, and rarely the heart, brain, and skeletal muscles. Dengue-associated liver involvement varies, ranging from asymptomatic transaminitis with an elevation of ALT or AST to fulminant acute liver failure, the latter of which carries a higher risk of mortality. Levels of transaminases (ALT and AST) were also found in previous studies to be significantly higher in severe dengue as compared to non-severe dengue [41–43]. This association was also found in our study as cases of severe dengue had significantly elevated levels of ALT and AST upon admission when compared to the non-severe dengue group.

Our study also showed that severe dengue patients had significantly deranged renal function parameters on admission as evidenced by higher urea and creatinine levels compared to non-severe dengue patients. Limited data from retrospective case series exhibited heterogeneity in the frequency of AKI associated with dengue. Using the Acute Kidney Injury Network (AKIN) criteria, a retrospective analysis in India reported an incidence of dengue-associated-AKI of 10.8% [44]. Another prospective cohort study conducted in India utilized the RIFLE criteria and reported an incidence of dengue-associated AKI of 35.7% [45]. AKI in dengue has been linked to direct cytopathic action by the virus itself on nephrons, or secondary to complications of hemodynamic instability (pre-renal AKI) or rhabdomyolysis [46].

LDH is an intracellular enzyme stored predominantly inside tissues or cells which may be released into the serum following injury. Raised serum LDH has been correlated with increased severity in other diseases such as severe sepsis, infections, and malignancies [47, 48]. In this study, the median LDH on admission was found to be significantly higher among the severe dengue cases compared to the non-severe group, implying a higher degree of DENV-induced cell or tissue injury in the former group. Our findings mirrored the findings of two previous studies by Perveen et al (Pakistan) and Sirikutt et al (Thailand) who reported that DHF patients had significantly higher levels of LDH on admission and throughout the hospitalization period, respectively [49, 50].

Shock and/or fluid accumulation was seen in severe dengue patients is believed to be caused by increased endothelial lining disruption leading to intractable leakage of intravascular albumin and other contents into the third space [51]. On admission, severe dengue patients in our study cohort were found to have significantly lower serum albumin levels as compared to the non-severe dengue group. This finding is similar to previous retrospective studies that found serum albumin to be a significant predictor for severe dengue, with levels of serum albumin inversely correlated with severity [52, 53]. A recent meta-analysis also highlighted hypoalbuminemia to be significantly associated with DSS [54].

### Clinical implications

Knowledge of the risk factors for severe dengue is important to clinicians during the triaging process. Results from our study suggest that dengue patients with hypertension may have higher odds of deterioration into severe dengue. Identification of this group of patients may aid in prioritizing needs for closer monitoring, including early pre-emptive referral to a tertiary hospital Although our study did not demonstrate a significant association with severe dengue for other comorbidities such as diabetes mellitus, obesity, or hyperlipidemia, a significant association has been reported in other studies.

### Strengths

Our study has several strengths. The dengue patients in our study were classified according to the latest WHO 2009 Dengue Classification Criteria that broadly categorized dengue patients into two groups, namely severe dengue, and non-severe dengue. Most previous studies were performed based on the older 1997 WHO Dengue Classification Criteria.

The format of our CRF was highly similar to the dengue admission form used during usual clinical practice. Therefore, although our study is retrospective, data collection approximated prospective data collection.

Extensive literature from the past three decades has highlighted a few established risk factors that may contribute to increased dengue severity such as age, female gender, secondary dengue infection, and dengue serotype. We controlled these factors by matching the cases and controls according to age (+/- 5 years old difference), gender, and date of hospital admission (+/- 2 weeks) to account for the seasonal variation of dengue serotypes.

We recorded data on the duration of fever at presentation as a surrogate marker of the time taken to seek treatment. In our study, the duration of fever at presentation was not significantly different between the cases and controls, suggesting that delays in seeking treatment are an unlikely confounding factor.

Few studies have attempted to investigate the relationship between obesity and severe dengue among adults, as many prior studies did not consider obesity as a comorbid condition.

### Limitations and future directions

Our study has several limitations. The reliability of our findings depended upon the accuracy of the information documented in the original patients’ notes. There may be variations in the type of data collected, especially in terms of signs and symptoms experienced by the patient. As highlighted earlier, this limitation may be reduced by the fact that patient information was recorded using a standardized dengue admission sheet very similar to our CRF.

We do not have data on patient-specific dengue serotype, as this is not routinely tested due to cost issues, adding a potential confounder. Nevertheless, we have attempted to address this issue by matching cases and controls according to their date of hospital admission, to increase the likelihood of both cases and controls being infected by the same serotype.

## Conclusion

In summary, our case-control study found that the odds of severe dengue were higher among patients with underlying hypertension, and lower in those presenting with headache or skin rash on admission. Lastly, severe dengue was again shown in our study to be associated with significant derangements in various hematological and biochemical laboratory parameters on admission. These observations may help to improve the triaging process.

## Supporting information

S1 Checklist (DOCX)

S1 File (DOCX)
