# Supplementary material for: A double whammy: The association between comorbidities and severe dengue among adult patients—A matched case-control study
Source: PLoS One. 2022 Sep 20;17(9):e0273071. doi: 10.1371/journal.pone.0273071 (PMC9488767; doi:10.1371/journal.pone.0273071)
Supplement: S1 File — (DOCX) [file pone.0273071.s002.docx]

# **Supporting Information**

## **List of Abbreviations**

| ACOR  CI  DENV  RNA  WHO  DF  DHF  DSS  HLA  NHMS  HSAJB  ICU  ELISA  MSK  URTI  Hb  Hct  WCC  ALT  AST  CK  LDH  CRF  cOR  MREC  IQR  CRP  NO  ET-1  Ang II  eNOS  EG  AKI | Adjusted conditional odds ratio  Confidence interval  Dengue virus  Ribonucleic acid  World Health Organization  Dengue fever  Dengue haemorrhagic fever  Dengue shock syndrome  Human Leukocyte Antigen  National Health and Morbidity Survey  Hospital Sultanah Aminah Johor Bahru  Intensive care unit  Enzyme-linked immunosorbent assay  Musculoskeletal  Upper respiratory tract infection  Haemoglobin  Haematocrit  White cell count  Alanine aminotransferase  Aspartate aminotransferase  Creatinine kinase  Lactate dehydrogenase  Case-report form  Conditional odds ratio  Medical Research and Ethics Committee  Interquartile range  C-Reactive protein  Nitric oxide  Endothelin 1  Angiotensin II  Endothelial nitric oxide synthase  Endothelial glycocalyx  Acute kidney injury |
| --- | --- |
